# Utility of Targeted Sequencing Compared to FISH for Detection of Chronic Lymphocytic Leukemia Copy Number Alterations

**DOI:** 10.3390/cancers16132450

**Published:** 2024-07-03

**Authors:** J. Erin Wiedmeier-Nutor, Chantal E. McCabe, Daniel R. O’Brien, Erik Jessen, Cecilia Bonolo de Campos, Nicholas J. Boddicker, Rosalie Griffin, Cristine Allmer, Kari G. Rabe, James R. Cerhan, Sameer A. Parikh, Neil E. Kay, Huihuang Yan, Daniel L. Van Dyke, Susan L. Slager, Esteban Braggio

**Affiliations:** 1 Division of Hematology and Oncology, Department of Medicine, Mayo Clinic, Phoenix, AZ 85054, USA; 2 Division of Computational Biology, Department of Quantitative Health Sciences, Mayo Clinic, Rochester, MN 55905, USA; 3 Division of Clinical Trials and Biostatistics, Department of Quantitative Health Sciences, Mayo Clinic, Rochester, MN 55905, USA; 4 Division of Epidemiology, Department of Quantitative Health Sciences, Mayo Clinic, Rochester, MN 55905, USA; 5 Division of Hematology, Department of Medicine, Mayo Clinic, Rochester, MN 55905, USA; 6 Department of Immunology, Mayo Clinic, Rochester, MN 55905, USA; 7 Department of Laboratory Medicine and Pathology, Mayo Clinic, Rochester, MN 55905, USA

**Keywords:** CLL, NGS, FISH

## Abstract

**Simple Summary:**

Chronic lymphocytic leukemia (CLL) is a cancer of the blood and bone marrow, commonly affecting older adults. Most CLL patients have an abnormal copy number of chromosomes that are used for risk stratification. Chromosomal copy number changes have been evaluated using a technique called Fluorescence in situ hybridization (FISH). Next-generation sequencing (NGS) is now common practice in many cancers and in addition to DNA mutations, can also identify genetic copy number alterations. Together, these could inform disease prognosis and guide decisions on treatment. This study evaluated NGS as an alternative method to FISH for detecting clinically relevant chromosomal copy number changes in CLL.

**Abstract:**

Chronic lymphocytic leukemia (CLL) is characterized by multiple copy number alterations (CNAs) and somatic mutations that are central to disease prognosis, risk stratification, and mechanisms of therapy resistance. Fluorescence in situ hybridization (FISH) panels are widely used in clinical applications as the gold standard for screening prognostic chromosomal abnormalities in CLL. DNA sequencing is an alternative approach to identifying CNAs but is not an established method for clinical CNA screening. We sequenced DNA from 509 individuals with CLL or monoclonal B-cell lymphocytosis (MBL), the precursor to CLL, using a targeted sequencing panel of 59 recurrently mutated genes in CLL and additional amplicons across regions affected by clinically relevant CNAs [i.e., del(17p), del(11q), del(13q), and trisomy 12]. We used the PatternCNV algorithm to call CNA and compared the concordance of calling clinically relevant CNAs by targeted sequencing to that of FISH. We found a high accuracy of calling CNAs via sequencing compared to FISH. With FISH as the gold standard, the specificity of targeted sequencing was >95%, sensitivity was >86%, positive predictive value was >90%, and negative predictive value was >84% across the clinically relevant CNAs. Using targeted sequencing, we were also able to identify other common CLL-associated CNAs, including del(6q), del(14q), and gain 8q, as well as complex karyotype, defined as the presence of 3 or more chromosomal abnormalities, in 26 patients. In a single and cost-effective assay that can be performed on stored DNA samples, targeted sequencing can simultaneously detect CNAs, somatic mutations, and complex karyotypes, which are all important prognostic features in CLL.

## 1. Introduction

Chronic lymphocytic leukemia (CLL) is characterized by multiple copy number alterations (CNAs) that are central to disease prognosis, risk stratification, and response to therapy [1,2,3,4]. Fluorescence in situ hybridization (FISH) is widely used in the clinical laboratory and is the gold standard for screening prognostic CNAs in CLL [1,4,5]. FISH panel assays, which typically detect 4–6 common recurring genetic defects, are relatively quick and have high specificity, but they require prior knowledge of the genetic abnormality to be screened and can detect only a limited number of alterations per assay [6]. Next-generation sequencing (NGS) has recently become more readily available for clinical applications and has the ability to screen the entire genome or multiple genes of interest in hematologic malignancies [7,8,9]. Furthermore, NGS has been used not only for mutation screening but also for identifying CNAs and structural variants, especially if whole genome sequencing (WGS) is used. Although WGS is the best sequencing approach for detecting CNAs, followed by whole exome sequencing (WES), both methods are still relatively expensive, and the analysis is bioinformatically more intensive compared to gene panels [6].

Targeted DNA sequencing is an alternative, less expensive, approach to identifying mutations, and is best for detecting small clones, subclones, and minimal residual disease [6]. Although targeted sequencing is not an established method for clinical CNA screening, it would be ideal to have a single clinical assay that could evaluate multiple relevant CNAs and mutations in a cost-effective manner. Hence, we evaluated the accuracy of using a custom sequencing panel for simultaneously detecting prognostic CNAs and gene mutations commonly tested in CLL in a large cohort of untreated patients with CLL and high-count monoclonal B-cell lymphocytosis (HC-MBL), the precursor to CLL [1,5,10,11,12,13,14].

## 2. Material and Methods

### 2.1. Patients

This cohort included treatment naïve CLL and HC-MBL patients from the Mayo Clinic CLL resource who were clinically seen in the Division of Hematology, Mayo Clinic. CLL and HC-MBL individuals were diagnosed between 2001 and 2019, had provided a blood specimen collected within two years of diagnosis for NGS, and had available FISH data (FISH assays were performed in Mayo Clinic Laboratories as previously described) [3] that was measured within 3 months of blood specimen used for NGS. Additional clinical characteristics obtained at the time of diagnosis included age, sex, Rai stage, serum β2 microglobulin levels, and immunoglobulin heavy chain variable region (*IGHV*) mutation status. *IGHV* mutational status was previously performed as the standard of care for using NGS in the Department of Laboratory Medicine and Pathology at Mayo Clinic. The CLL-International Prognostic Index (CLL-IPI), comprised of 5 individual clinical and leukemic prognostic factors, was computed as defined previously for each patient [15,16]. This research was approved by the Mayo Clinic Institutional Review Board, and all participants provided written informed consent.

### 2.2. DNA Sequencing

DNA was extracted from peripheral blood mononuclear cells (PBMC) if tumor purity (clonal B-cell, detected by flow cytometry) was ≥80%. If tumor purity was <80%, clonal B-cells were first enriched using magnetic beads for CD5+/CD19+. We designed a DNA targeted sequencing panel, covering exons of 59 recurrently mutated genes in CLL and additional amplicons across regions affected by clinically relevant CNAs, as previously described (Supplemental Figure S1) [13]. The median coverage depth per sample was 1799×, with 83% of those samples having a median depth of coverage >1000× per nucleotide, allowing for the detection of mutations with variant allelic fraction (VAF) as low as 1%. Somatic mutations were called using MuTect2 in tumor-only mode, and only high-impact mutations (frameshift, nonsense, and splicing variants) and missense mutations in CLL hot spots were selected [17].

### 2.3. Copy Number Alterations

We used the CNV-calling algorithm PatternCNV to detect commonly detected FISH abnormalities across the study cohort, which were sequenced in six different batches [18]. PatternCNV provides CNV estimates based on coverage and variability patterns. In brief, the algorithm divides each exon into bins where coverage is standardized across samples, CNVs are estimated in each bin, and multiple bins within the exon are smoothed, leading to a CNV maximum likelihood estimation. To correct for potential sequencing batch effects, PatternCNV was initially run to quantify exon coverage variation across the chromosomes without four clinical FISH abnormalities (i.e., all chromosomes except 11, 12, 13, and 17). We then used principal component analyses and correlation matrices to group the samples into distinct clusters (N = 4) that each showed similar exon coverage patterns. Samples in each of the four clusters were then independently re-run through PatternCNV using all chromosomes and FISH abnormalities, and the log_2_ ratio within each patient was median-centered within the cluster. For quality control, we determined the noisiness within each sample using the difference in the median absolute deviation (DiffMAD), and samples with a DiffMAD score >0.3 were excluded (examples of poor DiffMAD scores shown in Supplemental Figure S2). CNV analyses were blinded to clinical FISH results. Based on the CNV calls from targeted sequencing, we computed the complex karyotype, defined as ≥3 or more CNV abnormalities [19,20,21].

### 2.4. Statistical Methods

Concordance of FISH and targeted sequencing results were calculated using FISH as the gold standard (see Supplemental Methods). Discordances were further investigated by manual visual inspection of CNV plots and chromosomal microarray (CMA) on available samples. CMA assays were performed according to the manufacturer’s protocol and methods described in Baliakas et al. [22].

## 3. Results

### 3.1. Patient Characteristics

A total of 522 patients were sequenced with our targeted sequencing panel. Of those, 509 (379 CLL and 130 HC-MBL) had successful CNA calls and were included in the final analysis (Table 1); the remaining 13 (2%) had high DiffMAD scores (>0.3) and were excluded from further analyses. The median age of the final cohort was 62 years, 70.3% were male, and 49.8% (N = 242) were *IGHV* mutated. Based on 465 individuals with CLL-IPI prognostic scores, most individuals were categorized as low or intermediate risk at 37.4% (n = 174) and 34.0% (n = 158), respectively, whereas 20.6% (n = 96) and 8.0% (n = 37) were categorized as high and very high risk (Table 1).

### 3.2. Sensitivity, Specificity, Positive Predictive Value, and Negative Predictive Value Comparing FISH and Targeted Sequencing

**Figure 1:**
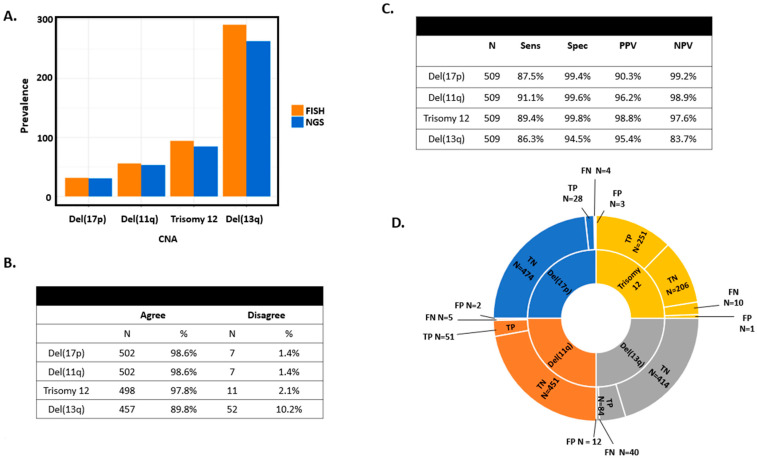
Descriptive results of CNA calling across four FISH abnormalities. (**A**) Bar graphs comparing detection of CNA by FISH or NGS techniques. (**B**) FISH and NGS concordance for del(17p), del(11q), trisomy 12, and del(13q). (**C**) Sensitivity, specificity, PPV, and NPV among CLL and MBL patients of CNA detection using FISH as the gold standard. (**D**) Sunburst representation of true positives (TP), true negatives (TN), false positives (FP), and false negatives (FN) of targeted sequencing using FISH as the gold standard for del(17p), del(11q), trisomy 12, and del(13q). Sens: sensitivity. Spec: specificity. PPV: positive predictive value. NPV: negative predictive value. FP: false positive. FN: false negative. TP: true positive. TN: true negative.

**Figure 2:**
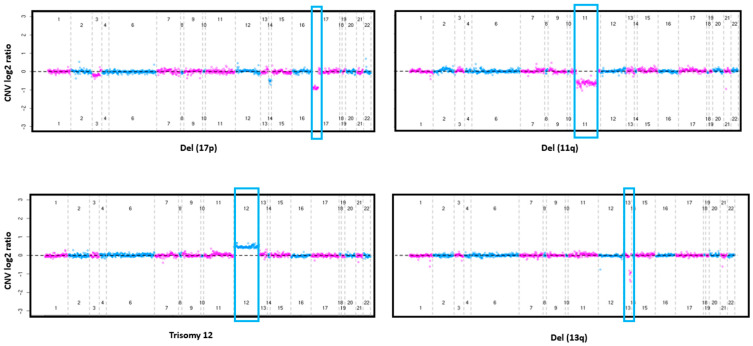
Plots showing chromosome abnormalities for del(17p) (**upper left**, blue box), del(11q) (**upper right**, blue box), trisomy 12 (**lower left**, blue box), and del(13q) (**lower right**, blue box). CNV log2 ratios are represented along the *y* axis and chromosomes are represented along the *x* axis. A CNV log 2 ratio of 0 corresponds to 2 copies. Values above and below 0 correspond to gains and losses, respectively. Each dot represents the respective targeted sequencing probe coverage.

From the FISH data analysis, we had a prevalence of del(17p) of 6.3% (n = 32), del(11q) of 11.0% (n = 56), trisomy 12 of 18.5% (n = 94), and del(13q) of 57.2% (n = 291) (Table 1, Figure 1A). Based on CNV analyses from targeted sequencing, we identified 31 (6.1%) individuals with del(17p), 53 (10.4%) with del(11q), 85 (16.7%) with trisomy 12, and 263 (51.7%) with del(13q) (Figure 1A). When we compared CNV analyses with clinical FISH data, we found a concordance of 98.6% for del(17p) and del(11q), 97.8% for trisomy 12, and 89.7% for del(13q) (Figure 1B). The sensitivity, specificity, PPV, and NPV for each of the clinically relevant FISH CNV are all elevated (Figure 1C,D), e.g., for del(17p), sensitivity was 87.5%, specificity was 99.4%, PPV was 90.3%, and NPV was 99.2% (Figure 1C,D). Del(13q) had the lowest concordance with a sensitivity of 85.3%, specificity of 94.5%, PPV of 95.4%, and NPV of 83.7%. CNV plots representing each chromosomal abnormality are shown in Figure 2.

### 3.3. Discordance between FISH and Targeted Sequencing

**Figure 3:**
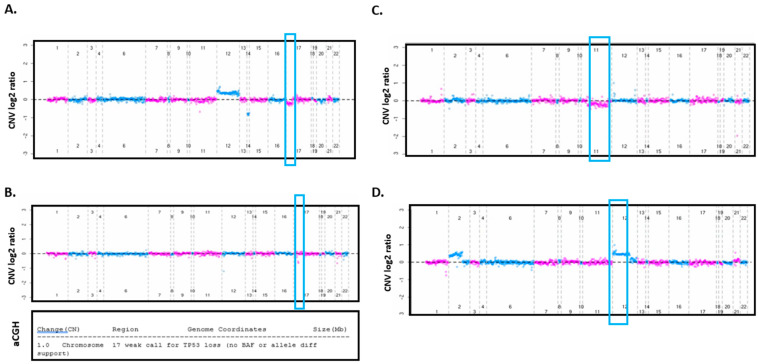
CNA plots of individuals who had discordant FISH and NGS results. (**A**) Example of discordant sample for del(17p) (targeted sequencing negative/FISH positive). Del(17p) is clearly seen on CNV plot (blue boxes); however, the deletion was below the CNV algorithm’s threshold. (**B**) Example of discordant CNA between targeted sequencing and FISH for del(17p). Microarray (CMA) result is shown in the middle graph and bottom panel. CMA confirmed del(17p) that was called by our CNV algorithm (blue box) but noted that it was a weak call for *TP53* loss. (**C**,**D**) Discordant CNAs between targeted sequencing and FISH for del(11q) and trisomy 12, respectively. CNAs were on the CNV plot but were not called by our algorithm.

A total of 77 samples were discordant between FISH and targeted sequencing (Figure 1B). For del(17p), we had four false-negative results from targeted sequencing (Figure 1D). We manually reviewed the CNV plots and identified three of the four individuals with clearly identifiable CNAs (example in Figure 3A). These false negatives were primarily due to the CNAs being slightly above our stringent log2 ratio threshold of −0.2 in our CNV algorithm. There were three individuals with false-positive results from sequencing for del17p (Figure 1D). We performed CMA on all three of these individuals. For one individual, CMA confirmed the presence of del17p from targeted sequencing results (Table 2, Figure 3B). We suspect that this deletion may have been too small for the FISH probe to identify, whereas targeted sequencing was sensitive enough to detect the deletion (Figure 3B). For the other two individuals, CMA did not show del(17p), which was consistent with FISH results.

For del(11q), five false-negative and two false-positive results were identified (Figure 1D). After a manual review of CNV plots, one false negative individual had a detectable CNA (Figure 3C). Only one of the remaining six discordant del(11q) individuals had a sample available for CMA, which confirmed the NGS finding of negative del11q (Table 2).

For trisomy 12, there were 10 false negatives and 1 false positive result (Figure 1D). After a manual review of CNV plots, two false negative individuals clearly had a CNA (Figure 3D). Only 1 of the remaining 9 discordant individuals had a sample available; CMA on that sample confirmed the NGS finding of positive trisomy 12 (Table 2).

The highest discordance, 52 individuals (40 false-negative and 12 false-positive results, Figure 1D), was found for del(13q). After a manual review of CNV plots, 9 false negative individuals clearly had a detectable CNA. A total of 9 of the remaining 43 discordant individuals had samples available for CMA where that approach confirmed the NGS findings in 5 individuals and aligned with FISH in the other 4 samples (Table 2). 

### 3.4. Other CNAs and Genetic Aberrations as Detected by Targeted Sequencing

**Figure 4:**
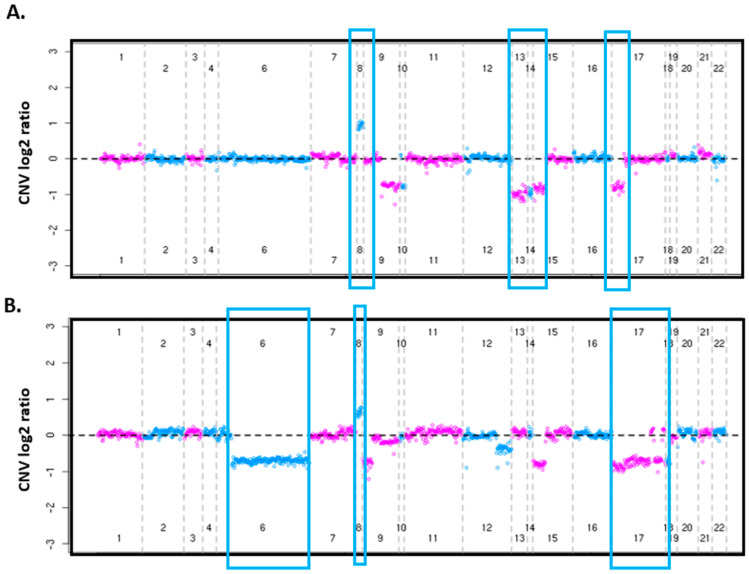
Identification of cases with complex karyotypes using targeted sequencing. (**A**) CNV plot showing deletions of chromosomes 9, 10, 13, 14, 15, and 17 and gain of chromosome 8 (blue boxes). (**B**) CNV plot showing deletions on chromosomes 6, 9, 12, 15, and 17 and gain of chromosome 8 (blue boxes).

Targeted sequencing identified other chromosomal aberrations, including the presence of complex karyotypes. Using our CNV detection method with targeted sequencing data, we were able to detect other known CNAs in CLL including del(6q) (n = 18, 3.5%), del(14q) (n = 11, 2.1%), gain 8q (n = 16, 3.1%), and gain 2p (n = 26, 5.1%). Using the CNV algorithm, we detected complex karyotypes in 26 samples (defined as ≥3 chromosomal abnormalities as per NCCN guidelines [1], Figure 4). Of these 26 samples, 10 were considered low to intermediate risk according to CLL-IPI.

As previously reported, the targeted sequencing panel also provided information on prognostically important somatic mutated genes. In the current cohort, 338 (66.4%) individuals had a mutation in at least one recurrently mutated gene in CLL, and 177 (34.8%) individuals had two or more. We found that 58 (11.4%) individuals harbored *TP53* mutations, and 22 (4.9%) individuals harbored both *TP53* mutations and del(17p) [13].

## 4. Discussion

FISH panel testing is widely used in clinical practice to identify prognostic chromosomal abnormalities in CLL. However, comprehensive genetic profiling has become increasingly important in the workup of hematologic malignancies providing additional clinically relevant genetic alterations. Here, we showed that targeted sequencing can infer prognostic CNAs while also providing additional clinically relevant genetic alterations in CLL patients.

When evaluating possible reasons for false negatives by NGS, our study supports the need for manual visual inspection of negative CNV calls that are slightly below the threshold of CNV calling to identify additional CNVs. Across the 4 FISH abnormalities on which we focused for this study, we recovered 15 out of the 59 false negatives that were clearly CNV calls on manual inspection. We found that del(13q) had the highest number of false negatives (N = 40). We suspect this is due to our sequencing panel design, as we used fewer NGS probes in the miRNA region targeted by the FISH panel. To address this, we are increasing probe coverage for the next version of our sequencing panel.

Genomic arrays are an alternative method for identifying CNAs [23]. We evaluated possible reasons for false positives with NGS using CMA. We found that discordance between FISH and targeted NGS may be due to the higher sensitivity of targeted sequencing (possibly due to the small sizes of the FISH probe), as targeted sequencing was able to detect some CNAs that FISH missed.

Importantly, targeted sequencing provided mutation results across 59 genes, of which FISH and genomic arrays cannot evaluate. Mutation analysis included *TP53,* which is one of the most clinically important adverse prognostic markers in CLL. Patients with any *TP53* abnormality, including del(17p), have shorter progression-free survival (PFS) and overall survival (OS) and are less responsive to traditional chemoimmunotherapy used in CLL [24,25]. Our group and others have shown that having multi-allelic *TP53* aberrations is associated with a significantly shorter time to first treatment and OS compared to single *TP53* aberration and wild-type *TP53* [26]. Additional genes included in the panel, such as *SF3B1*, *NOTCH1*, *BIRC3*, and *EGR2*, have also been associated with differential prognosis and time and response to therapy in multiple studies [27,28,29,30].

Targeted sequencing also provided complex karyotype status, which is known to be associated with inferior outcomes [19,20,21]. Herein, we identified 10 of the 26 individuals with complex karyotype as high-risk for poor prognosis who were otherwise considered low to intermediate-risk according to the CLL-IPI model risk categorization alone.

In summary, using a targeted sequencing panel, one can capture multiple prognostic and predictive genomic alterations with high resolution in a single assay. We showed a high concordance using bioinformatic methods that allow for accurate detection of prognostic CNAs when comparing targeted sequencing with FISH. Furthermore, it proved to be a robust tool for identifying cases with complex karyotypes, which is becoming very valuable as an adverse prognostic factor for CLL prognostication [31]. This assay also allowed the comprehensive screening of *TP53* alterations (deletion and mutation), placing patients in the high-risk CLL-IPI group. Collectively, the approach allowed us to better define the genetic makeup of individuals, including those not previously identified as high-risk, giving us valuable prognostic information on outcomes.

It is important to note that we are not proposing targeted NGS as the ideal method for the detection of adverse mutations and CNAs in every circumstance (for example, turn-around time for targeted sequencing may take one to two weeks, whereas FISH may be available in a few days), but rather it is a cost-effect NGS tool that can provide additional prognostic information [6]. Our group and others have reported on the prognostic significance of multiallelic *TP53* aberrations; however, results from other studies have been mixed, and thus the significance of detecting somatic mutations in addition to CNAs in CLL has not definitively been defined [32,33,34]. This study also highlights that molecular techniques for identifying CNAs have pitfalls and can produce false-negative and/or false-positive results. Future studies to test the value of targeted NGS are warranted to validate our findings. Future directions will include a comparison of targeted NGS and FISH in relapsed/refractory CLL patient samples, as these samples would be expected to carry a greater number of molecular aberrations, which may not be detectable by traditional FISH assays.

## 5. Conclusions

Genetic aberrations characterize disease, define risk stratification, impact therapeutic decision-making, and can be used to monitor disease. In CLL, prognostic CNAs have historically been identified using FISH. Targeted NGS, however, has the ability to identify somatic mutations, in addition to large structural alterations, and thus may be used in characterizing the molecular profile of CLL. Despite the advantages of targeted sequencing, the issues regarding false negatives and false positives will need to be addressed in future studies.

## Figures and Tables

**Table 1:** Clinical and demographic characteristics.

	CLL/SLL (N = 379)	MBL (N = 130)	Total (N = 509)
**Age at Diagnosis**			
Missing	0	0	0
Mean (SD)	61.8 (10.4)	64.2 (9.9)	62.4 (10.3)
Median	61.4	64.0	62.0
Q1, Q3	55.0, 69.0	58.0, 72.0	55.0, 70.0
Range	(31.0–89.0)	(42.0–86.0)	(31.0–89.0)
**Gender**			
F	105 (27.7%)	46 (35.4%)	151 (29.7%)
M	274 (72.3%)	84 (64.6%)	358 (70.3%)
**CLL-IPI**			
Missing	27	17	44
Low risk (0–1)	109 (31.0%)	65 (57.5%)	174 (37.4%)
Intermediate (2–3)	127 (36.1%)	31 (27.4%)	158 (34.0%)
High (4–6)	81 (23.0%)	15 (13.3%)	96 (20.6%)
Very high (7–10)	35 (9.9%)	2 (1.8%)	37 (8.0%)
**Rai Category**			
Missing	1	0	1
Rai 0: Low risk	287 (75.9%)	130 (100.0%)	417 (82.1%)
Rai 1 or 2: Intermediate risk	59 (15.6%)	0 (0.0%)	59 (11.6%)
Rai 3 or 4: High risk	32 (8.5%)	0 (0.0%)	32 (6.3%)
**B2M**			
Missing	12	3	15
Mean (SD)	3.2 (1.9)	2.4 (1.2)	3.0 (1.8)
Median	2.6	2.0	2.4
Q1, Q3	2.0, 3.5	1.7, 2.6	1.9, 3.3
Range	(1.1–16.2)	(1.2–9.9)	(1.1–16.2)
**IGHV**			
Missing	11	12	23
Mutated	158 (42.9%)	84 (71.2%)	242 (49.8%)
Unmutated	210 (57.1%)	34 (28.8%)	244 (50.2%)
**FISH del(17p)**			
Normal	350 (92.3%)	127 (97.7%)	477 (93.7%)
Abnormal	29 (7.7%)	3 (2.3%)	32 (6.3%)
**FISH del(11q)**			
Normal	328 (86.5%)	125 (96.2%)	453 (89.0%)
Abnormal	51 (13.5%)	5 (3.8%)	56 (11.0%)
**FISH del(13q)**			
Normal	151 (39.8%)	67 (51.5%)	218 (42.8%)
Abnormal	228 (60.2%)	63 (48.5%)	291 (57.2%)
**FISH Trisomy 12**			
Normal	312 (82.3%)	103 (79.2%)	415 (81.5%)
Abnormal	67 (17.7%)	27 (20.8%)	94 (18.5%)

**Table 2:** Discordant samples detected by our CNV algorithm were further evaluated by CMA. CNAs are listed in the left column, sample numbers in the middle column, and microarray results in the right column. Samples highlighted in grey are concordant between targeted sequencing and CMA.

Del(17p)	Sample #	Microarray Result	Notes
**NGS+/FISH−**	**2238**	**absent**	**Tetraploid**
	**3046**	**absent**	
	**3283**	**present**	**subclonaldel(17p)**
**Del(11q)**			
**NGS−/FISH+**	**1856**	**absent**	
**Trisomy 12**			
**NGS+/FISH−**	**WC2760**	**present**	
**Del(13q)**			
**NGS−/FISH+**	**1370**	**absent**	
	**1446**	**present**	**Del(13q) is centromeric of miRNAs**
	**3091**	**absent**	
	**3670**	**present**	**small del(13q)—865 kb**
	**3968**	**present**	**small del(13q)—718 kb**
	**WC2760**	**absent**	
**NGS+/FISH−**	**2433**	**present**	
	**2513**	**present**	
	**3046**	**absent**	

Grey highlights congruent CNV and CMA results. **#** is ‘Number’.

## Data Availability

Data to support the findings of this study can be requested from the corresponding authors.

## Supplementary Materials

The following supporting information can be downloaded at: https://www.mdpi.com/article/10.3390/cancers16132450/s1, Figure S1: schematic overview of research methodology (created with Biorender); Figure S2: two examples showing samples with high DiffMADD scores that were excluded from further analysis. (A) DiffMADD score of 0.529 and (B) DiffMADD score of 0.308; and Supplemental Methods.
