# Supplementary material for: Utility of Targeted Sequencing Compared to FISH for Detection of Chronic Lymphocytic Leukemia Copy Number Alterations
Source: Cancers (Basel). 2024 Jul 3;16(13):2450. doi: 10.3390/cancers16132450 (PMC11240685; doi:10.3390/cancers16132450)
Supplement: Supplementary file 1 [file cancers-16-02450-s001.zip › cancers-3050853-supplementary.pdf]

Supplemental Methods – Statistical Methods

Using FISH as the gold standard, sensitivity for each FISH abnormality was calculated as true positives (i.e., targeted sequencing positive for specific CNV abnormality and FISH positive samples) divided by the number of FISH positive results for the specific abnormality. Specificity for each FISH abnormality was calculated as true negative (i.e., targeted sequencing negative for CNV and FISH negative) divided by the number of FISH negative results for the specific abnormality. Positive predictive value (PPV) for each abnormality was calculated as true positive divided by the number of targeted sequencing positive results for the specific abnormality. Negative predictive value (NPV) for each FISH abnormality was calculated as true negative divided by the number of targeted sequencing negative results for the specific abnormality.

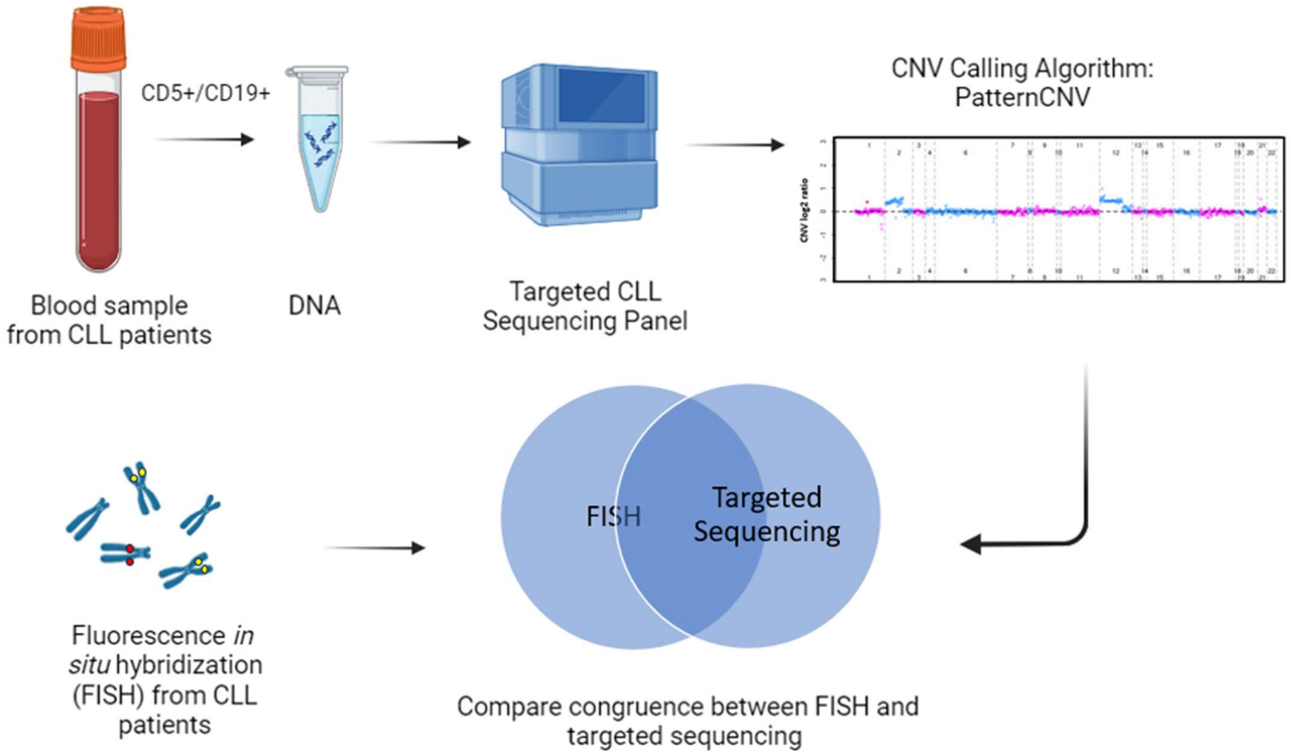

Supplemental Figure S1. Schematic overview of research methodology (created with Biorender).

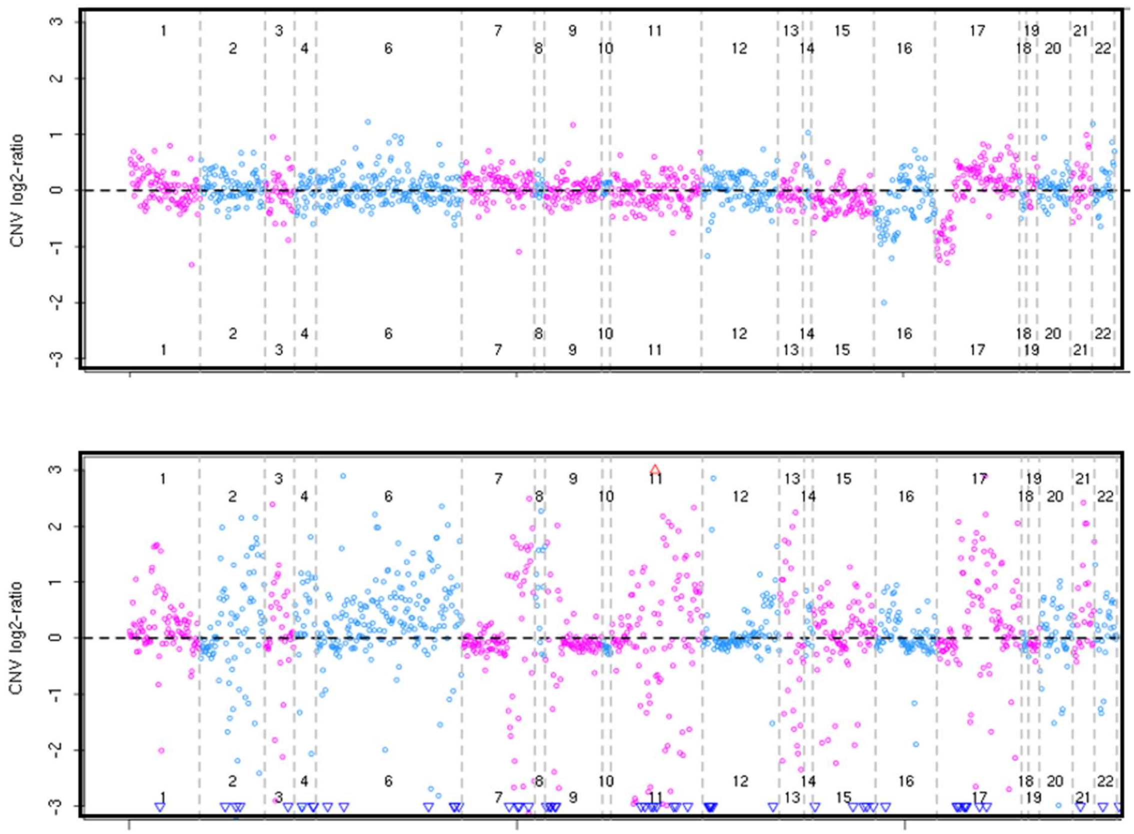

Supplemental Figure S2. Two examples showing samples with high DiffMADD scores that were excluded from further analysis. (A) DiffMADD score of 0.529 and (B) DiffMADD score of 0.308.
